# Origin of the Co‐Seismic Variations of Elastic Properties in the Crust: Insight From the Laboratory

**DOI:** 10.1029/2021GL093619

**Published:** 2021-06-22

**Authors:** F. Paglialunga, F. X. Passelègue, M. Acosta, M. Violay

**Affiliations:** ^1^ École Polytechnique Fédérale de Lausanne LEMR Lausanne Switzerland

**Keywords:** damage zone, dynamic rupture, faulting, friction, seismic properties

## Abstract

Seismological observations highlighted that earthquakes are often followed by changes in elastic properties around the fault zone. Here, we studied the origin of these variations using stick‐slip experiments on saw‐cut granite samples presenting different degrees of bulk damage (i.e., microcracks). Stick‐slip events were induced under triaxial compression configuration with continuous active ultrasonic measurements at confining pressures representative of upper crustal conditions (15–120 MPa). Both the P‐wave velocity ($V_{P}$) and amplitude ($A_{P}$) showed drops, concurrently with stress drops, and had a non‐monotonic dependence toward the fault's stress state. Our experimental results suggest that co‐seismic changes in $V_{P}$ were mostly controlled by the elastic re‐opening of microcracks in the bulk, rather than by co‐seismic damage or the formation of fault gouge. Co‐seismic changes in $A_{P}$ were controlled by a combination of elastic re‐opening of microcracks in the bulk and inelastic processes (i.e., co‐seismic damage and gouge formation and dilation).

## Key Points

- Laboratory earthquakes are associated with seismic velocity and amplitude drops in crustal rocks
- Velocity drops are controlled by the stress acting on the fault and degree of damage in the fault wall
- Amplitude drops are partly controlled by the state of stress and partly affected by dissipative (inelastic) phenomena occurring on‐fault

## 1 Introduction

It is known that during the failure of intact rock specimens, the formation and propagation of microcracks in the bulk increases up to failure, inducing a continuous reduction in seismic velocity (Lockner et al., 1977). If this behavior reflected the failure of natural faults, monitoring the evolution of seismic velocity could help in detecting possible earthquake preparation phases. Earthquakes, effectively, correspond to the brittle failure of the upper crust due to, in the majority of cases, stress accumulation along crustal faults resulting from long‐term tectonic loading. Seismological observations highlighted that earthquakes are associated with co‐seismic changes in elastic properties around the fault zone (Brenguier et al., 2008; Chen et al., 2010; Froment et al., 2013; Hobiger et al., 2012; Qiu et al., 2020; Wegler et al., 2009; Wu et al., 2016). Most of these studies showed co‐seismic velocity variations occurring predominantly in the shallow part of the crust (4–5 km depth). The attributed origin is not unique, and could involve different physical models (Rubinstein & Beroza, 2004): (a) co‐seismic damage caused by ground motion, (b) pore pressure variations, (c) microcracks response to static stress change, or (d) fault damage zone response to fault motion. Indeed, in the upper crust, faults are composed by a fault core, where most of the slip occurs, and by a zone of damage surrounding the fault core (Caine et al., 1996; Faulkner et al., 2010; Lockner et al., 2009; Rempe et al., 2013; Wallace & Morris, 1986). While we can have access to direct measurements of the damage zone's width close to the surface (ranging in between few meters and few kilometers), we do not have direct measurements of its evolution with depth, apart from specific drilling projects, which highlight that both the damage zone and fault core are very narrow at depth (Holdsworth et al., 2011). Such observations are supported by a recent numerical study (Okubo et al., 2019), highlighting that the size of the damage zone generated by earthquake ruptures is maximum close to the surface and decreases with depth. Because of that, the response of fault zones to loading in terms of seismic properties is expected to vary spatially and temporally, and to be a function of both fault structure and travel paths of the seismic waves (Nishizawa, 1982).

To get insights on co‐seismic seismic properties variations throughout the seismic cycle, several experimental studies focused at monitoring the evolution of elastic properties through laboratory friction experiments on artificial faults (Kendall & Tabor, 1971). Yoshioka and Iwasa (2006) already used transmission waves to monitor a brass fault contact evolution under normal and shear stress, finding a clear increase in wave amplitude with the increase of normal and shear stresses and amplitude variations linked with precursory slip due to change of the fault's contact area. Following studies performed with gouge interfaces (Kaproth & Marone, 2013; Scuderi et al., 2016; Tinti et al., 2016) showed both co‐seismic and precursory changes in P‐wave velocity associated with laboratory earthquakes, attributed to the gouge layer dilation and its change of porosity. Scuderi et al. (2016) explored the complete spectrum of failure modes, from slow to fast earthquakes, showing that not only co‐seismic changes but also precursory variations of P‐wave velocity occur for each mode of failure. Fukuyama et al. (2018) studied amplitude variation during high‐velocity friction experiments. Moreover, Shreedharan et al. (2021) showed clear precursory P‐wave amplitude variations occurring with the instability nucleation phase and precursory P‐wave velocity variations distorted by the presence of the surrounding bulk material. These observations suggest that the elastic properties of the bulk material surrounding the fault may play a role in seismic velocity drops associated to natural earthquakes, as well as its recovery in the months following the rupture. Indeed, seismic waves velocities are sensitive to a change in the degree of damage (i.e., presence of microcracks) of the medium they travel through (Blake et al., 2013; Brantut, 2015; Griffiths et al., 2018; Guéguen & Palciauskas, 1994; Kuttruff, 2012; Nasseri et al., 2009; Nishizawa, 1982).

Our study aims at understanding how much of the change in seismic properties observed during earthquakes is controlled by co‐seismic damage occurring on‐ (i.e., gouge production) and off‐ (i.e., formation of microcracks in the fault wall due to seismic rupture) fault, and how much is instead affected by the presence of the initial degree of damage characterizing the bulk material and its response to stress changes. To this end, we conducted stick‐slip experiments (Brace & Byerlee, 1966) under a wide range of confining pressures on granite saw‐cut cylindrical samples presenting two different degrees of initial bulk damage, to mimic different fault damage zone properties.

## 2 Experimental Methods

### 2.1 Materials

The tested material is La Peyratte granite, a crustal rock presenting a modal composition of 38.5% plagioclase, 28.5% quartz, 20% K‐Feldspar, 13% biotite with an average grain size of 800 µm.

Right‐circular cylindrical samples were prepared with 38 mm diameter and 78 mm height. Some were thermally treated before the experiments by slowly heating them (5°C/min, to avoid thermal gradients inside the sample (Wang et al., 2013)) up to different target temperatures (650°C) and let cool down to ambient temperature inside the oven overnight, to avoid thermal shock. Target temperatures were chosen above the α‐β quartz transition (572°C), allowing intense intra‐granular cracking, randomly oriented in the bulk, producing isotropically damaged media (Glover et al., 1995; Pimienta et al., 2019; Wang et al., 2013), with reduced fracture toughness (Kang et al., 2020; Nasseri et al., 2007). To characterize the different samples, density and porosity were measured, obtaining densities of 2.63 g/cm^3^ and 2.58 g/cm^3^ and porosities of 0.4% and 6.6%, respectively, for non‐treated and thermally treated granite at 650°C.

**Figure 1:**
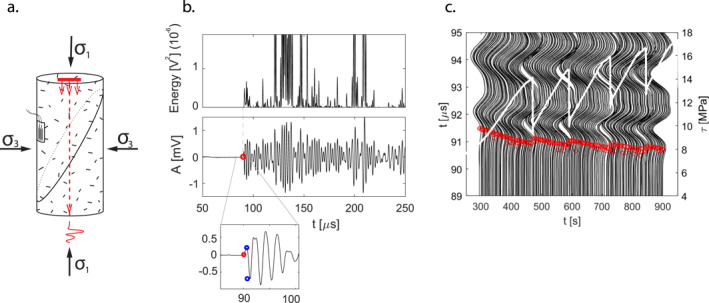
(a) Sample configuration with applied external loads $\sigma_{1},\;\sigma_{3},\;$pulsing direction (in red) and strain gauge location. (b) P‐wave arrival time detection; the top panel displays the wave energy evolution with time, the bottom panel displays the detected P‐wave arrival time (in red), and P‐wave first arrival amplitude (inset, in blue). (c) Seismic waves evolution during a stick‐slips series performed for a treated sample at a $P_{C}$ of 15 MPa. Red markers indicate the arrival time detected by the automatic picking. Shown waves are sampled (1:5). In white, the shear stress evolution during the test.

Samples were saw‐cut with an orientation of 30° to the vertical axis, creating an artificial fault plane. The fault roughness was imposed by hand using #240 grit sandpaper, generating a smooth fault, optimally oriented for reactivation, avoiding the propagation of new secondary fractures in the surrounding medium (Renard et al., 2020). The lack of secondary fracture formation under this configuration has been verified in previous experimental work (e.g., Acosta et al. [2019]'s supporting information). A strain gauge was glued on the sample at an intermediate distance between the fault and the sample edge, measuring the axial deformation of the bulk material (Figure 1a).

### 2.2 Testing Procedure

Tests were run in an oil medium high‐pressure triaxial apparatus, FIRST (installed at LEMR, EPFL). The samples were first submitted to a target confining pressure ($P_{C}$) (15, 30, 45, 60, 90, and 120 MPa), with a subsequent increase of axial load. Axial load was applied by controlling the oil flow rate (0.25 or 0.50 ml/min in few cases), pushing the piston, generating a displacement rate of ∼6 · 10^−6^ mm/s. For the different samples (non‐treated and treated), experiments were conducted starting from the lowest $P_{C}$ and, once the stick‐slips series was performed, $P_{C}$ was increased to the following target $P_{C}$ and a new stick‐slips series performed, up to the highest target $P_{C}$. Two displacement transducers were placed beside the sample, measuring locally the sample’ shortening and/or the fault slip. Mechanical data were recorded at a frequency of 100 Hz for the whole duration of the tests.

### 2.3 Acoustic Measurements

Active acoustic measurements were recorded during deformation, using acoustic sensors (PZT crystal) placed inside the top and bottom anvils of the triaxial apparatus, with a recording frequency of 100 Hz. The acquisition system setup and the picking procedure were modified and adapted from Acosta and Violay (2020) (refer to the supporting information for details).

Seismic waveforms were used to measure the evolution of P‐wave velocity and amplitude along the experiment. Once detected the P‐wave arrival time ($t_{P}$) the P‐wave velocity ($V_{P})$ was computed as

(1) $$V_{P}=\frac{L_{\text{corrected}}}{t_{P}}$$

with $L_{\text{corrected}}$ the length of the sample, systematically corrected by the elastic shortening and slip occurring. The P‐wave amplitude ($A_{P}$) was computed as the difference in amplitude between the first maximum value and minimum value of the P‐wave (Figure 1b, inset). Seismic measurements were performed in the vertical direction, parallel to the sample axis (ray path showing the largest variations in wave velocity due to the mechanical anisotropy occurring during differential loading) (refer to the Supporting Information, Figure S1).

## 3 Experimental Results

**Figure 2:**
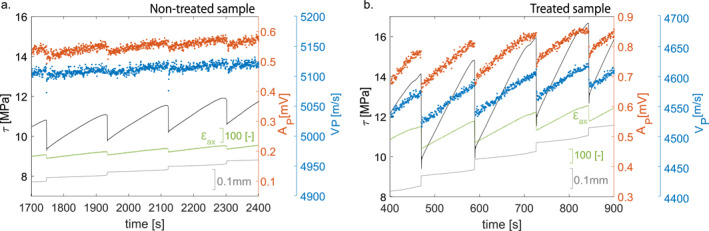
Evolution of shear stress (black), fault slip (gray), axial strain (light green), *V*_P_ (blue), and *A*_P_ (orange) with time during instabilities at $P_{C}=$15 MPa for non‐treated (a) and treated sample (b).

Stick‐slip experiments conducted under different $P_{C}$ were used to investigate seismic properties evolution throughout the seismic cycle. For each of them, the shear stress increased first linearly and, once reached the fault strength, dropped to a residual value (Figure 2). As expected, the higher the applied $P_{C}$, the higher the fault strength, stress drop, and resulting slip were observed. Concerning the seismic properties, an increase of $V_{P}$ and $A_{P}$ was observed during the hydrostatic loading up to the target $P_{C}$. Moreover, both $V_{P}$ and $A_{P}$ responded to the applied differential stress accordingly, increasing during loading and decreasing during unloading. For both the non‐treated and treated sample, the increase in $V_{P}$ during differential loading ($IV_{P}^{loading}$) was larger for low $P_{C}$, and smaller for high $P_{C}$ (Figure S2). In particular for the non‐treated sample, $IV_{P}^{loading}$ (from $(\sigma_{1}-\sigma_{3})=0$ to the fault's strength) was ∼200 m/s, ∼140 m/s, ∼90 m/s, ∼80 m/s, respectively at a $P_{C}\;$of 15, 30, 60, and 90 MPa. For the treated sample, $IV_{P}^{loading}$ was ∼390 m/s, ∼220 m/s, ∼150 m/s, respectively at a $P_{C}\;$of 15, 45, and 120 MPa. $A_{P}$ changed in a similar way during differential loading ($IA_{P}^{loading}$) for the different $P_{C}$. $IA_{P}^{loading}$ for the non‐treated sample was ∼3.3 × $10^{-4}$ V, ∼1.9  ×$10^{-4}\;$V, ∼1.0 × $10^{-4}$ V, respectively at a $P_{C}\;$of 15, 30, 60, and 90 MPa. For the treated sample, $IA_{P}^{loading}$ was ∼4.5 × $10^{-4}$ V, ∼4.55 × $10^{-4}$ V, and ∼1.2 × $10^{-4}$ V, respectively at a $P_{C}\;$of 15, 45, and 120 MPa. As stress drops occurred, associated to seismic fault slip, a drop in $V_{P}$ as well as in $A_{P}$ was observed.

**Figure 3:**
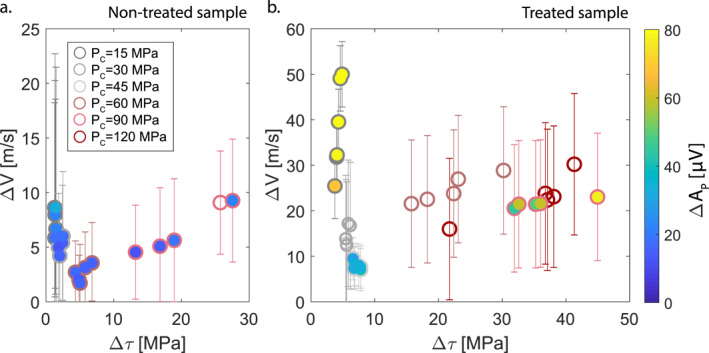
P‐wave velocity drops ($\Delta V_{P}$) evolution with associated shear stress acting on the fault measured during stick‐slips for the different $P_{C}\;$for on‐treated (a) and treated (b) sample. Error bars indicate the error related to the velocity drop estimation. The observed $\Delta V_{P}$ correspond in percentage to a range of 0.03%–0.35% and 0.16%–1.23%, respectively, for the non‐treated and treated sample. Color bar indicates associated P‐wave amplitude drops ($\Delta A_{P}$). Empty symbols are for cases in which it was not possible to measure $\Delta A_{P}$.

These co‐seismic drops in velocity ($\Delta V_{P}$) and amplitude ($\Delta A_{P}$) were computed for each stick‐slip, and compared with their respective stress conditions (Figure 3). $\Delta V_{P}$ did not show a linear dependence on stress conditions applied to the fault (i.e., normal stress, confining pressure, and shear stress). In the case of non‐treated sample, for low $P_{C}$ (15–30 MPa), hence for low Δτ (∼1–3 MPa), $\Delta V_{P}$ were larger (∼4–9 m/s) than for events recorded at higher $P_{C}\;$(60 MPa) and medium Δτ (∼4–9 MPa), which were ∼2–6 m/s. For higher $P_{C}$ (90 MPa) and the highest Δτ (∼12–28 MPa), $\Delta V_{P}$ increased again (∼4–9 m/s). The same trend was observed for the treated sample, but with much larger $\Delta V_{P}$. For low $P_{C}$ (15 MPa), hence for low Δτ (∼3–4 MPa), $\Delta V_{P}$ were larger (∼25–50 m/s) than for events recorded at higher $P_{C}\;$(30–45 MPa), hence for medium Δτ (∼4–9 MPa), which were ∼6–18 m/s. For higher $P_{C}$ (60–90–120 MPa), and the highest Δτ (∼15–45 MPa), $\Delta V_{P}$ increased again (∼15–30 m/s). Overall, a large difference in magnitude was noted between the non‐treated and the treated sample (Figure 2): the latter showed larger increases during elastic loading and larger drops for similar stress drops. $\Delta A_{P}$ evolution with stress conditions is similar to $\Delta V_{P}$ evolution, with higher values for low$\;P_{C}$ and high $P_{C}$ and lower values for intermediate $P_{C}$, for both the non‐treated and the treated samples (Figure 3 in color bar).

## 4 Discussion

In our experiments, a non‐monotonic $\Delta V_{P}$ evolution with shear stress drops was observed (Figure 3), suggesting that distinct physical processes coexist at the origin of velocity changes during stick‐slip instabilities, due to the combination of initial bulk damage and loading conditions. In particular, these drops in velocity during stick‐slip events could be related to (i) horizontal microcracks re‐opening in the bulk, after initial closure during increasing differential stress (Passelègue et al., 2018), due to differential stress reduction or (ii) co‐seismic damage induced around the fault during dynamic rupture propagation and fault motion (Marty et al., 2019; Okubo et al., 2019).

To test these hypotheses, we estimated the maximum possible contribution of microcracks re‐opening due to co‐seismic stress drop on the associated $\Delta V_{P}$. Such effect is expected to be similar during both loading and unloading of the bulk (if no adhesion is considered on the microcrack, for example, stress‐induced microcrack opening/closure is a reversible process). Under these assumptions, $IV_{P}^{loading}$ for each $P_{C}$ can be used to estimate the contribution of microcracks opening following co‐seismic stress drops and associated strain release, not considering possible co‐seismic damage occurring off‐fault. We predicted $\Delta V_{P}\;$only due to the re‐opening of microcracks occurring in the bulk as follows:

(2) $$\Delta V_{P}^{predicted}=\frac{\;\Delta \;\varepsilon_{ax}}{I\varepsilon_{ax}^{loading}}\;\cdot \;IV_{P}^{loading}$$

where $\Delta \varepsilon_{ax}$ is the drop in axial strain measured concurrently with stress drop, $I\varepsilon_{ax}^{loading}$ the increase in axial strain during differential loading (strain gauge located in the bulk material, far enough from the fault, expected to capture elastic deformation of the bulk).

**Figure 4:**
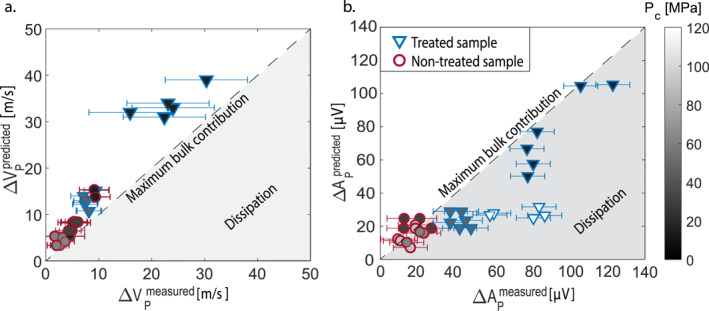
(a) $\Delta V_{P}^{predicted}\;\text{vs.}\;\Delta V_{\;P}^{measured}$ are shown for non‐treated (in burgundy) and treated (in blue) samples. The dashed line represents the 1:1 slope which divides the plot in two regions: (in white) domain where $\Delta V_{P}$ are completely explained by elastic re‐opening of the microcracks present in the bulk (in gray) dissipative domain, where $\Delta V_{P}\;$are explained by dissipative phenomena like co‐seismic damage and gouge shearing. (b) $\Delta A_{P}^{predicted}\;\text{vs}\;\cdot \;\Delta A_{P}^{measured}$ are shown for non‐treated (in burgundy) and treated (in blue) samples. The black line divides the plot in two regions: (in white) domain where $\Delta A_{P}$ are completely explained by elastic re‐opening of the microcracks present in the bulk (in gray) dissipative domain, where $\Delta A_{P}\;$are explained by dissipative (inelastic) phenomena (i.e., shearing of the gouge layer).

$\Delta V_{P}^{predicted}$ for all the events at each Pc for both treated and non‐treated samples, showed the same evolution with loading conditions of the ones experimentally observed $\left(\Delta V_{P}^{measured}\right)$. In fact, by plotting them together (Figure 4a), a linear dependence between the two is noted, with a slope very close to 1:1, indicating that $\Delta V_{P}^{measured}$ are well explained by the co‐seismic re‐opening of microcracks in the bulk, resulting from the release of strain. This suggests that in our experimental configuration, no significant co‐seismic damage was generated during rupture propagation, or it was negligible with respect to the observed velocity variations. Once again, the non‐monotonic trend observed as a function of applied stress (Figure 3) is explained by the interplay between $P_{C}$ and $IV_{P}^{loading}$. For low $P_{C}$ the induced stress drops are very small (∼1–3 MPa/∼3–4 MPa, respectively, for non‐treated and treated sample) but the related $IV_{P}^{loading}\;$(seen here as the maximum potential velocity drop caused by microcracks opening, at a specific $P_{C}$) is very large (∼200/∼390 m/s), generating high velocity drops (∼4–9/∼7–50 m/s). On the contrary, for medium $P_{C}$ the induced stress drops are a bit higher (∼4–9 MPa), but the corresponding $IV_{P}^{loading}\;$ is lower (∼140/∼210 m/s), generating quite small velocity drops (∼2–6/∼6–18 m/s), while for high $P_{C}$ the observed stress drops are very large (∼12–28/∼15–45 MPa) and even if the related $IV_{P}^{loading}\;$is very small (∼80 m/s/∼150 m/s), the resulting velocity drops are larger (∼4–9/∼15–30 m/s) (refer to Supporting Information and Figure S4 for a conceptual scheme).

Remarkably, while co‐seismic $\Delta V_{P}$ proved, in our experiments, to be mostly related to the re‐opening of microcracks in the bulk and elastic relaxation, we could observe some gouge production on the post mortem samples fault surfaces (in particular on the one of the treated sample), which is expected to have an influence on the seismic properties measured across the sample (Scuderi et al., 2016; Shreedharan et al., 2021; Tinti et al., 2016). In particular, the presence of a gouge layer is expected to affect $A_{P}$, considered as a simplified way to account for attenuation (Lockner et al., 1977) (i.e., the higher the amplitude of the wave, the lower the attenuation and vice versa). Compared to $V_{P}$, which is mainly affected by elastic processes such as microcracks closure and re‐opening, $A_{P}$ is also influenced by the fault's specific stiffness and by the inelastic, dissipative deformation processes occurring on and off‐fault (i.e., frictional sliding of microcracks in the bulk and/or gouge particle shearing).

A prediction similar to the one described above (Equation 2) was tempted to test if $A_{P}\;$measured in these experiments was also mainly influenced by the bulk properties and stress conditions. Equation 2 was modified and $V_{P}$ was replaced by $A_{P}$ as follows:

(3) $$\Delta A_{P}^{predicted}=\frac{\Delta \varepsilon_{ax}}{I\varepsilon_{ax}^{loading}}\;\cdot \;IA_{P}^{loading}$$

with $\Delta A_{P}^{predicted}$ the predicted amplitude drops and $IA_{P}^{loading}$ the overall increase of $A_{P}$ during differential loading. For the non‐treated sample, the prediction works well, with values falling very close to the prediction line of slope 1:1 (Figure 4b). However, for the treated sample, this is true only for the lower $P_{C}\;$(15 MPa). For higher $P_{C}$ (45 and 120 MPa), the predicted drops do not mimic the measured ones, the latter being notably larger (up to 400% larger). This might be explained by the change in the fault's contacts and/or by non‐elastic processes occurring either in the bulk (i.e., friction caused by shear along microcracks) or on the fault surfaces (i.e., gouge production, shearing, and dilation). Since the expected stress responsible for microcracks shearing is larger than the one expected to activate shearing along the artificial fault, we assume that the non‐elastic processes observed are caused by the fault's response to stick‐slips. This was verified by analyzing the evolution of $A_{P}$ with cumulative slip (Figure S3), since (a) gouge production is expected to increase linearly with cumulative fault slip (Archard, 1953) and (b) slip requires shearing of gouge particles under high applied stresses. Given that (a) the thermally treated sample is expected to have a lower fracture toughness than the non‐treated one (Nasseri et al., 2007), (b) we observed a decrease in $A_{P}\;$for consecutive stick‐slips only under medium to high $P_{C}\;$(i.e., normal load acting on the fault), and (c) that we could observe a large amount of gouge on the post‐mortem sample's fault, we ascribed $A_{P}\;$behavior to be a function of the gouge production (Frérot et al., 2018) and subsequent gouge particles shearing during fault's slip under these conditions. This looks coherent with the evolution of the fault specific stiffness (Pyrak‐Nolte et al., 1990) for the different stress conditions (Figure S5), which in the case of the treated sample reaches a sort of saturation for the highest $P_{C}$ (120 MPa)(i.e., the gouge once filled all the voids available in the interface and compacted, will not deform any further for higher$\;P_{C}$, not influencing $k_{F}$ ).

## 5 Implications and Conclusions

Summarizing our interpretation of the results, co‐seismic $\Delta V_{P}$ seem to be controlled by the combination of bulk properties and applied stress (i.e., re‐opening of the microcracks present in the bulk concurrently with stress drop). This does not imply that other phenomena occurring during stick‐slips, such as gouge layer dilation, could not contribute to $\Delta V_{P}$ itself, but only that their influence, compared to one of the pre‐existing microcracks in the bulk, resulted negligible in our experiments. In addition, while co‐seismic $\Delta A_{P}$ also looked to be controlled by the combination of bulk properties and applied stress when the presence of gouge was not dominant, they were probably controlled by dissipative processes occurring on‐fault when the conditions (treated sample and higher applied stress) allowed an important production of gouge, hence a necessary shearing of gouge particles.

Conversely to previous experimental studies (Kaproth & Marone, 2013; Scuderi et al., 2016; Shreedharan et al., 2021), no significant and clear precursory variation of seismic velocity and amplitude was observed. This might be due to several reasons; among the others, the localized nature of the nucleation phenomenon, known to be the cause of observed pre‐seismic slip. Depending on the nucleation patch size, either a low or a high‐stress perturbation will be induced in its vicinity. A nucleation patch length significantly smaller than fault length is expected under this configuration (Harbord et al., 2017). Assuming this, the stress release during the nucleation of instability is expected to affect only a small fraction of the whole sample, without inducing any strong premonitory change in $V_{P}$ or $A_{P}$. Another reason could be related to our resolution of the seismic measurements, which may be not high enough to capture precursory changes which remain lost within the error linked to the present measurements.

However, our results could help to better understand in which conditions precursory variations of seismic properties can actually be detected and used to monitor fault's state of stress. It is clear that the wallrock's elastic properties have a huge control on the seismic properties measured across the system. It was recently shown that this distortion is crucial for observations of the aforementioned precursory phase (Shreedharan et al., 2021). For this reason, the luckiest combination to observe this variation would be to encounter a fault composed by a wide gouge layer and with a large nucleation patch. The shearing of gouge particles within the fault zone will strongly affect the seismic amplitude, which is the parameter the most sensitive to inelastic processes.

Moreover, even if a direct comparison remains risky given the differences in the applied conditions and the large uncertainties in the estimation, there are some analogies between the relative variations in $V_{P}$ recorded in this study and the ones observed after real earthquakes. It emerges that the overall range of values measured in our experiments (0.03%–0.35% and 0.16%–1.23% respectively for non‐treated and treated sample), performed under stress conditions representative of the upper crust, is comparable to the ranges of values measured after real earthquakes (Brenguier et al. [2008] estimate variations of ∼0.02%–0.07%, Chen et al. [2010] find ∼0.04%–0.08%, Nimiya et al. [2017] find ∼0.4%–0.8%, Qiu et al. [2020] find ∼0.15%–0.25%, Taylor and Hillers [2020] find ∼0.15%). The similarity between our observations and the ones referring to natural earthquakes, suggests that the monitored seismic properties could be controlled by the same factors (i.e., combination of propagation of seismic waves through fault core, damage zone, wallrock). In fact, measurements performed across artificial gouge faults, monitoring $V_{P}$ evolution of the only gouge layer with no contribution of the surrounding medium, showed much higher relative $V_{P}$ variations ∼1%–4% (Scuderi et al., 2016; Tinti et al., 2016) (for a graphical representation refer to the Supporting Information, Figure S8).

Finally, given the impossibility to measure natural seismic variations of the only fault core, monitoring the evolution of seismic velocity along faults surrounded by large damage zones, could be of interest for observing co‐seismic changes during shallow earthquakes, since the combination of large and highly damage zones and low‐stress conditions lead to an extremely high sensitivity in velocity changes due to stress perturbations, especially at low depths. Moreover, since many earthquakes are preceded by a nucleation stage (Latour et al., 2013; Ohnaka, 2003; Ruiz et al., 2014; Socquet et al., 2017; Tape et al., 2018), which is expected to release part of the stress along the fault, the amplitude evolution may provide, under the aforementioned conditions, some indications about stress evolution along the fault and the proximity to failure. This kind of observations could, yet, be limited by the current spatial resolution of seismological observations and by the knowledge of the damage zones in seismogenic faults.

## Supporting information

Supporting Information S1

## Data Availability

The raw data can be found at the following address: http://doi.org/10.5281/zenodo.4892328.
