# Supplementary material for: Origin of the Co‐Seismic Variations of Elastic Properties in the Crust: Insight From the Laboratory
Source: Geophys Res Lett. 2021 Jun 22;48(12):e2021GL093619. doi: 10.1029/2021GL093619 (PMC8365675; doi:10.1029/2021GL093619)
Supplement: Supplementary file 1 — Supporting Information S1 [file GRL-48-e2021GL093619-s001.pdf]

AGU PUBLICATIONS

Geophysical Research Letters

Supporting Information for

Origin of the co-seismic variations of elastic properties in the crust: new insight from the laboratory

F. Paglialunga[1], F. X. Passelègue[1], M. Acosta[1], M. Violay[1]

[1]École Polytechnique Fédérale de Lausanne, LEMR, Lausanne, Switzerland

Contents of this file

Text S1 to S7
Figures S1 to S8

Introduction

This supplementary material contains details on the methods used in this article and supplementary material for the discussion and interpretation of the data. Supplementary items 1-6 contain supplementary text and supplementary figures.

P-wave monitoring in multiple directions.

During our experiments, the sample is subjected to several confining pressures. While hydrostatic loading to a target Pc is expected to close microcracks in an isotropic manner, differential loading is expected to close further the cracks oriented perpendicularly to the loading direction (Nishizawa 1982; Nur 1971), but to open cracks oriented parallel to the loading direction, creating an anisotropic crack distribution. In this regard, the seismic properties were measured in the vertical direction, parallel to the sample axis. This ray path shows the largest variations in wave velocity due to the mechanical anisotropy occurring during differential loading and subsequent stick-slip events.

This was checked through a test performed monitoring seismic waveforms in multiple directions. V_{P} was computed along four directions (0°, 21°, 38°, 50° with respect to the horizontal direction). For all directions, an increase in Vp was observed during both hydrostatic pressure increase and differential stress increase. Moreover, despite a slight difference between them, the increase in velocity looks almost comparable in magnitude. This suggests that the bulk material in the sample remains in a pretty elastic domain, and that the stress needed to induce microcracks opening and propagation is higher than the stress needed to make the macroscopic fault slip. Hence, it looks reasonable, in case of impossibility to monitor at high recording frequency seismic waves in multiple directions (as the present case), to monitor seismic waves only in the direction affected the most by mechanical anisotropy.

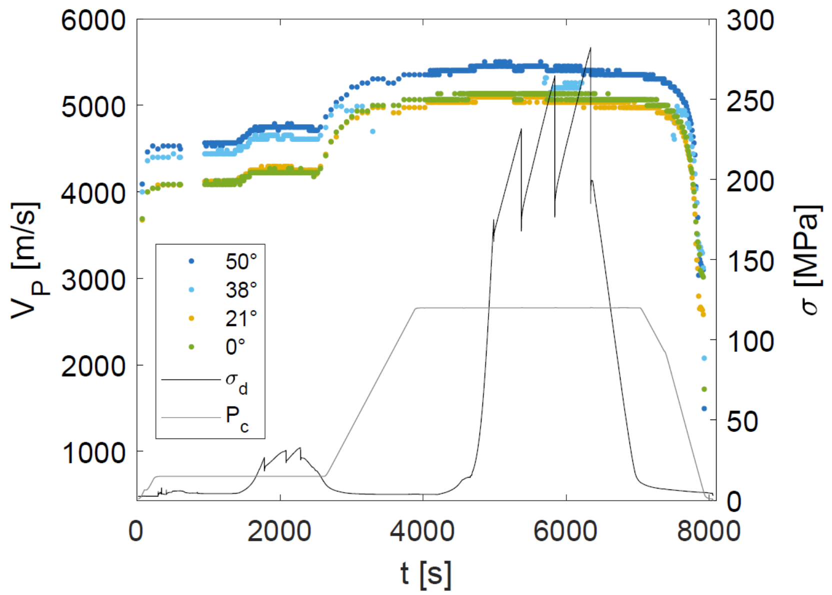

Figure S1. Evolution of confining pressure (Pc) and differential stress ($\boldsymbol{\sigma_d}$) respectively in gray and black. Evolution of $\boldsymbol{V_P}$ for the different monitoring directions for the whole duration of the test.

Experimental setup.

In our experimental conditions, increasing the axial stress led to an increase of both shear and normal stresses acting along the fault interface (equations (1,2)):

$$\sigma_n = \frac{\sigma_1+\sigma_3}{2} - \frac{\sigma_1-\sigma_3}{2}\cos(2\theta) \quad (1)$$

$$\tau = \frac{\sigma_1-\sigma_3}{2}\, sin(2\theta) \quad (2)$$

with θ angle between the fault plane and the applied vertical stress, and σ_1, and σ_3 the axial and radial stress respectively (Figure 1a). When the maximum fault's strength was reached, instabilities occurred along artificial fault (i.e. laboratory earthquakes (Brace and Byerlee 1966)).

Acoustic measurements.

Active acoustic measurements were recorded during deformation, using acoustic sensors (PZT crystal) placed inside the top and bottom anvils of the triaxial apparatus. The acquisition system setup and the picking procedure were modified and adapted from Acosta and Violay (2020). Acoustic signals were pulsed through OPMUX 12.0 ultrasonic multiplexer from the top P sensor and waves were received on the respective bottom one. The pulse voltage used was 300 V and the period of 0.5 µs. The recording frequency of each output wave was 200 MHz. The system allowed to monitor the evolution of the seismic velocity during the experiments at a frequency of 100Hz.

Once the waveforms were low-pass filtered (using a Butterworth filter with a cutoff frequency of 1 MHz), they were used to detect the P-wave arrival time (t_{P}). The cumulative integral of the squared wave amplitude was computed using trapezoidal method (unit spacing), obtaining a proxy of the energy the wave carries along. To get the sensitivity to change of this proxy of energy, its time derivative was computed. As the derivative reached a given threshold, t_{P} was returned (Figure 1b). Since the microcrack densities affecting the wave amplitude depend on the applied confining pressure, the threshold was systematically set, for the different applied $\mathrm{P_C}$, by manually peaking the one corresponding to the P-wave arrival. The measured t_{P} was then corrected by the P-wave travel time through top and bottom metal anvils. The initial length of the sample was corrected by both the elastic shortening of the bulk and by eventual fault slip ($L_{corrected}$), allowing the estimation of the P-wave velocity (V_{P}) as $V_{\mathrm{P}} = \frac{L_{\mathrm{corrected}}}{t_{\mathrm{P}}}$.

Besides the estimation of V_{P}, the seismic waveforms were used to measure the amplitude of the first P-wave arrival (Figure 1b), considered as a proxy for wave attenuation (Lockner, Walsh, and Byerlee 1977). Once t_{P} was detected, the first maximum and minimum of the P-wave were located and the amplitude (A_{P}) computed as the difference between them (Figure 1b, inset).

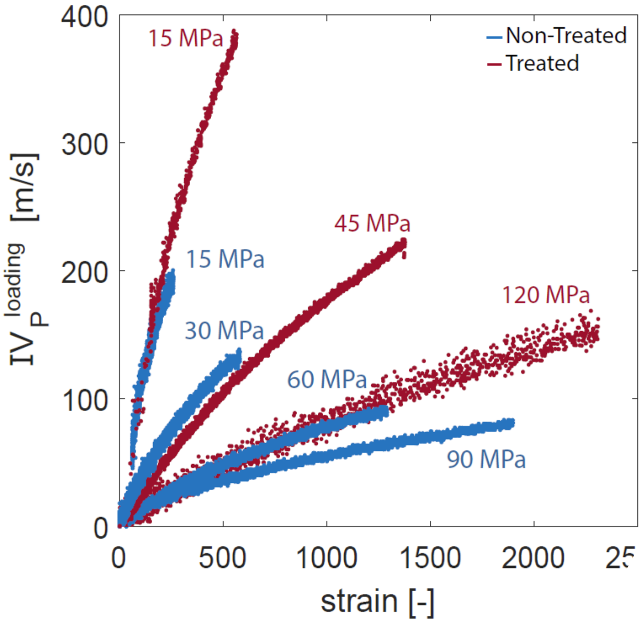

Figure S2. Evolution of V_{P} with axial strain during differential loading for the different P_{C}, for non-treated (in blue) and treated (in burgundy) samples.

Strain evolution.

The strain gauge placed in the central part pf the sample (Figure 1a), measured the axial strain evolution for the whole duration of the test.

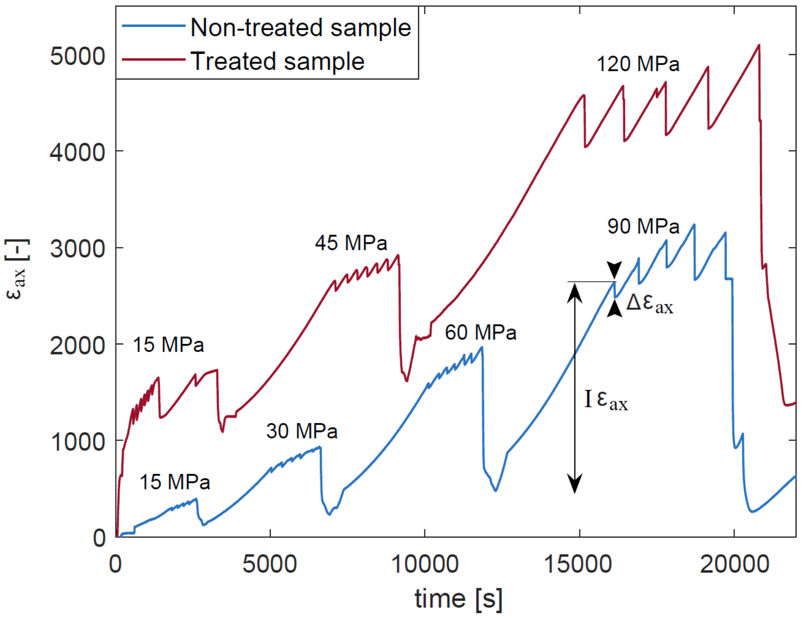

Figure S3. Evolution of axial strain during differential loading for the different $\boldsymbol{P_C}$, for non-treated (in blue) and treated (in burgundy) samples.

Interplay between $\boldsymbol{IV_P}$ and $\boldsymbol{\Delta\tau}$.

To have a clearer picture of the interplay between IV_P and $\Delta\tau$ in the definition of the observed velocity drops ΔV_P, both IV_P and $\Delta\tau$ were plotted for increasing P_C. One can observe that, for a range of low P_C, IV_P is the largest. Given the low P_C, many microcracks present in the bulk will still be open, hence the applied differential loading will produce a large IV_P (increase maximized by the monitoring direction parallel to the applied load and perpendicular to the horizontal cracks). For larger rages of P_C, the applied pressure will

have already closed most of the microcracks (but not all of them), hence IV_P will be smaller, but still appreciable (~150 m/s and ~80m/s respectively for treated and non-treated sample). At the same time, $\Delta\tau$ also evolve with P_C, increasing with increasing P_C. Summarizing, at low P_C, ΔV_P are described by preexisting bulk damage and are quite large because the related IV_P at low P_C is high. At higher P_C, IV_P is much lower, but on the other hand $\Delta\tau$ are much larger, so the strain released in the bulk (re-opening of preexisting microcracks) will be still quite high. At intermediate P_C, we have the smallest observed ΔV_P because both IV_P and $\Delta\tau$ are relatively small.

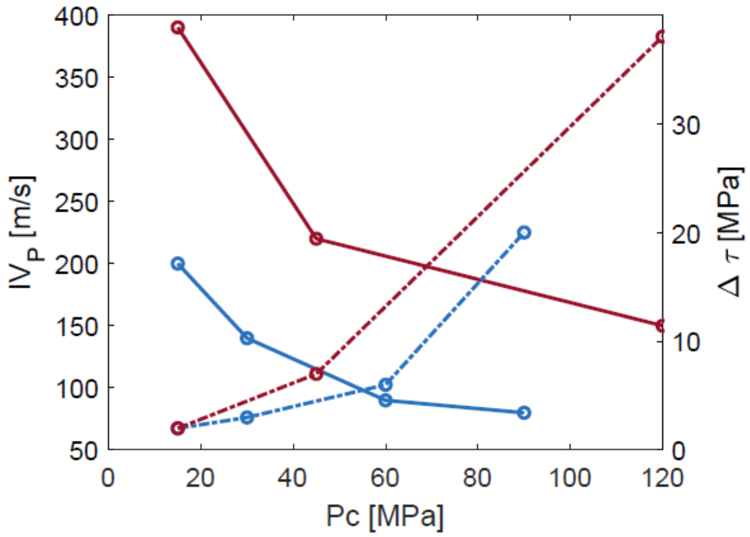

Figure S4. Evolution of $\boldsymbol{IV_P}$ (solid line) and $\boldsymbol{\Delta\tau}$ (dashed line) for increasing $\boldsymbol{P_C}$, for non-treated (blue) and treated (burgandy) samples. For $\boldsymbol{\Delta\tau}$ only a mean value is plot.

P-wave amplitude evolution with slip.

In the case of the non-treated sample, A_{P} increased for consecutive stick-slips (hence with cumulative slip) in a severe manner for the lowest P_{C} (30 MPa), in less severe manner for the medium and higher P_{C} (60 and 90 MPa) (Figure S3). In the case of the treated sample, A_{P} increased for consecutive stick-slips in a severe manner for the lowest P_{C} (15 MPa), while it slightly decreased for a medium P_{C} (45 MPa) and it clearly decreased for the highest P_{C} (120 MPa) up to reaching values even lower than the ones previous to the stick-slip series (i.e. negative $\mathrm{I}A_{\mathrm{P}}^{loading}$). The A_{P} behavior of the non-treated sample and of the treated sample at low P_{C} looked coherent with the observed evolution of the applied differential load, following the strain hardening profile (i.e. gradual increase of the peak strain over the stick-slip series) (dashed black curves in Figure S3). The A_{P} decrease with cumulative slip for higher P_{C} of the treated sample looked to be related to other phenomena, since its evolution deviates from the strain hardening tendency. It is well known from the theory of contact that wear production depends on several parameters (Frérot, Aghababaei, and Molinari 2018), among the many, some such as the applied load, the slip (Archard 1953; Scholz 1987) and fracture toughness (Fleming and Suh 1977).

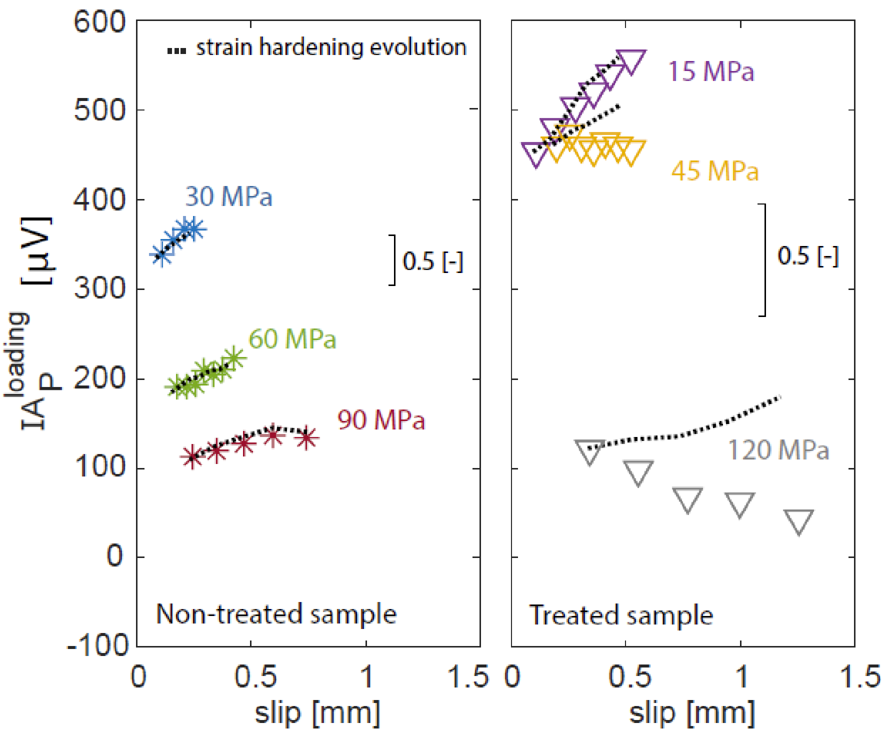

Figure S5. Evolution of A_P with slip during differential loading and stick-slips for the different P_C, for non-treated (on the left) and treated (on the right) samples. The dashed black line indicates the strain hardening curve, obtained by considering the strain increase within the stick-slip series, normalized by $I\varepsilon_{ax}^{loading}$.

Estimation of fault stiffness K_F.

The fault interface, acting as a displacement discontinuity, is able to absorb part of the energy carried by the wave, thus decreasing its amplitude. By increasing the applied stress, the contacts between the fault's surfaces will be stiffer, and the amplitude transmitted across will be larger. The fault stiffness was computed following (Pyrak-Nolte, Myer, and Cook 1990) as $k_{\mathrm{F}} = \frac{\partial \sigma_d}{\partial u_F}$ where σ_d is the differential stress acting on the fault and u_F the fault displacement, which describes the elastic deformation (ε_f) occurring on the contact asperities and voids or filling material. To obtain the latter, a correction of the deformation measured vertical LVDT (ε_{LVDT}) was corrected by the deformation of the apparatus (ε_{App}) and the deformation of the bulk material surrounding the fault (ε_{bulk}) measured through strain gauges following:

$$\varepsilon_f = \varepsilon_{LVDT} - \varepsilon_{bulk} - \varepsilon_{App}$$

$$\varepsilon_f = u_{ax}/L - \varepsilon_{bulk} - \Delta\sigma_d/E_{app}$$

Where u_{ax} is the displacement measured by the LVDTs, L is the sample's length, E_{app} is the deformation modulus of the apparatus. Once obtained the fault deformation, the effective displacement was obtained by multiplying by the sample length.

We expected, for increasing P_{C}, to observe higher k_{F} values. However, this happened only for the non-treated sample. For the treated sample, k_{F} considerably increased for the lower P_{C}, and increased much less for the highest P_{C} (120 MPa). This sort of k_{F} saturation can be related to the aforementioned relevant production of gouge, which, once filled all the voids available in the interface and compacted, will not deform any further for higher P_{C}, not influencing k_{F}.

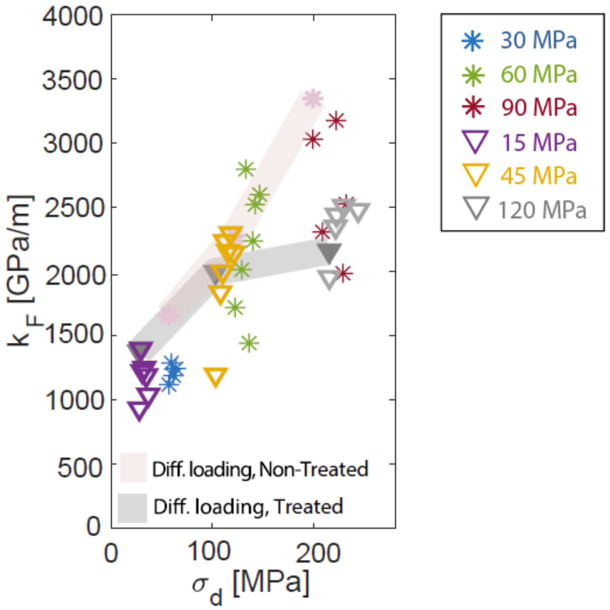

Figure S6. k_{F} evolution for different P_{C} for non-treated and treated samples. In shaded light / dark grey the k_{F} evolution during differential loading for the different P_{C} for non-treated / treated sample. Markers show k_{F} measured during the 'stick' phase of each of the observed events for different P_{C}.

Hydrostatic compression test.

Two hydrostatic compression tests on non-faulted samples have been performed, to capture V_P and A_P change during microcracks elastic closure (Figure S5). As expected (Blake, Faulkner, and Rietbrock 2013), a first non-linear large increase in V_P is observed, up to *crack closure pressure* (Birch 1961; Kern 1978), after which V_P increased linearly with applied stress. However, V_P did not increase in the same way for the two samples. In the non-treated sample, V_P increased of ~900 m/s over the whole range of confining pressures, while in the treated sample V_P increased of ~2500 m/s. Note that at the maximum reached $\mathrm{P_C}$ (160MPa) the two V_P still differed by ~500 m/s. A similar behavior was observed for A_P. A first non-linear large increase in A_P is observed, after which A_P increased linearly with applied stress.

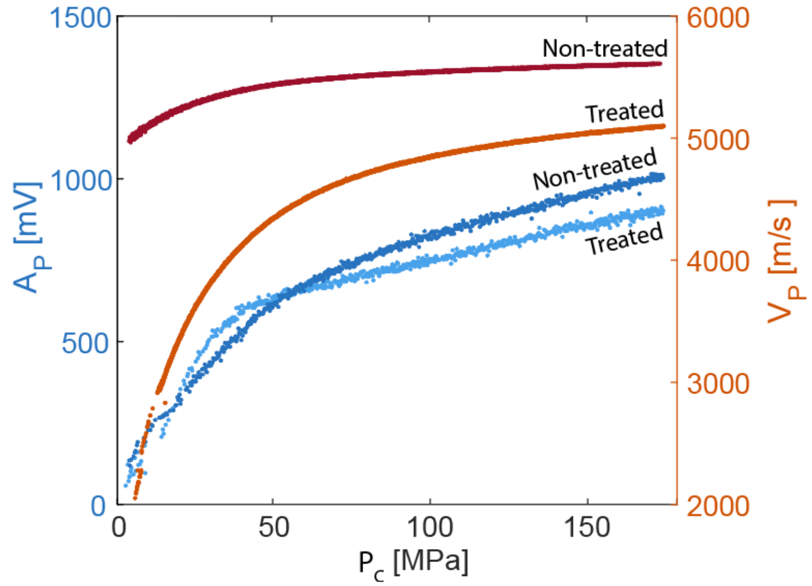

Figure S7. Evolution of V_P (in red) and A_P (in blue) for non-treated (dark color) and treated (light color) samples, for increasing P_C, during a hydrostatic compression test.

Comparison of observed velocity variations to natural observations.

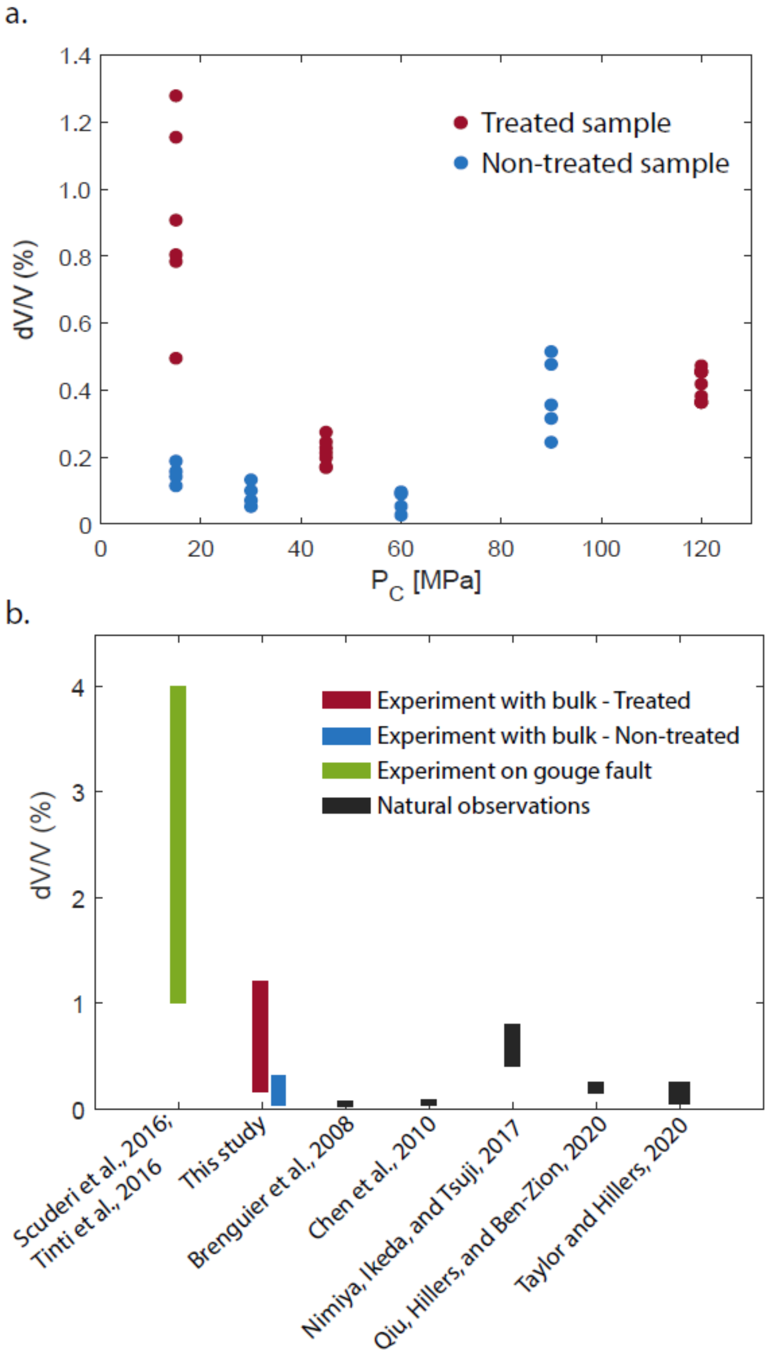

Figure S8. a. P-wave relative velocity drops (dV/V) evolution for the different P_C for non-treated (in blue) and treated (in burgundy) sample, with V the P-wave velocity measured

right before instability. b. Qualitative plot comparing velocity variations measured in the present work with the different ranges observed in both laboratory experiments (Scuderi et al. 2016; Tinti et al. 2016) and natural earthquakes (Brenguier et al. 2008; Chen et al. 2010; Nimiya, Ikeda, and Tsuji 2017; Qiu, Hillers, and Ben-Zion 2020; Taylor and Hillers 2020).

References

Acosta, Mateo and Marie Violay. 2020. "Mechanical and Hydraulic Transport Properties of Transverse-Isotropic Gneiss Deformed under Deep Reservoir Stress and Pressure Conditions." *International Journal of Rock Mechanics and Mining Sciences* 130(104235).

Archard, J. F. 1953. "Contact and Rubbing of Flat Surfaces." *Journal of Applied Physics* 24(8):981–88.

Birch, Francis. 1961. "The Velocity of Compressional Waves in Rocks to 10 Kilobars: 2." *Journal of Geophysical Research* 66(7):2199–2224.

Blake, O. O., D. R. Faulkner, and A. Rietbrock. 2013. "The Effect of Varying Damage History in Crystalline Rocks on the P- and S-Wave Velocity under Hydrostatic Confining Pressure." *Pure and Applied Geophysics* 170(4):493–505.

Brace, W. F. and J. D. Byerlee. 1966. "Stick-Slip as a Mechanism for Earthquakes." *Science* 153(3739):990–92.

Brenguier, F., M. Campillo, C. Hadziioannou, N. M. Shapiro, R. M. Nadeau, and E. Larose. 2008. "Postseismic Relaxation along the San Andreas Fault at Parkfield from Continuos Seismological Observations." *Science* 321(5895):1478–81.

Chen, Jiu Hui, Brnice Froment, Qi Yuan Liu, and Michel Campillo. 2010. "Distribution of Seismic Wave Speed Changes Associated with the 12 May 2008 Mw 7.9 Wenchuan Earthquake." *Geophysical Research Letters* 37(18):2008–11.

Fleming, J. R. and N. P. Suh. 1977. "The Relationship between Crack Propagation Rates and Wear Rates." *Wear* 44(1):57–64.

Frérot, Lucas, Ramin Aghababaei, and Jean François Molinari. 2018. "A Mechanistic Understanding of the Wear Coefficient: From Single to Multiple Asperities Contact." *Journal of the Mechanics and Physics of Solids* 114:172–84.

Kern, H. 1978. "The Effect of High Temperature and High Confining Pressure on Compressional Wave Velocities in Quartz-Bearing and Quartz-Free Igneous and Metamorphic Rocks." *Tectonophysics* 44(1–4):185–203.

Lockner, D. A., J. B. Walsh, and J. D. Byerlee. 1977. "Changes in Seismic Velocity and

Attenuation during Deformation of Granite." *Journal of Geophysical Research* 82(33):5374–78.

Nimiya, Hiro, Tatsunori Ikeda, and Takeshi Tsuji. 2017. "Spatial and Temporal Seismic Velocity Changes on Kyushu Island during the 2016 Kumamoto Earthquake." *Science Advances* 3(11).

Nishizawa, Osamu. 1982. "Seismic Velocity Anisotropy in a Medium Containing Oriented Cracks-Transversely Isotropic Case." *Journal of Physics of the Earth* 30(4):331–47.

Nur, Amos. 1971. "Effects of Stress on Velocity Anisotropy." *Sciences, Planetary* 76(8):2022–34.

Pyrak-Nolte, L. J., L. R. Myer, and N. G. W. Cook. 1990. "Transmission of Seismic Waves across Single Natural Fractures." *Journal of Geophysical Research* 95(B6):8617–38.

Qiu, Hongrui, Gregor Hillers, and Yehuda Ben-Zion. 2020. "Temporal Changes of Seismic Velocities in the San Jacinto Fault Zone Associated with the 2016 M w 5.2 Borrego Springs Earthquake." *Geophysical Journal International* 220(3):1536–54.

Scholz, Christopher H. 1987. "Wear and Gouge Formation in Brittle Faulting." *Geology* 15(6):493–95.

Scuderi, M. M., C. Marone, E. Tinti, G. Di Stefano, and C. Collettini. 2016. "Precursory Changes in Seismic Velocity for the Spectrum of Earthquake Failure Modes." *Nature Geoscience* 9(9):695–700.

Taylor, G. and G. Hillers. 2020. "Estimating Temporal Changes in Seismic Velocity Using a Markov Chain Monte Carlo Approach." *Geophysical Journal International* 220(3):1791–1803.

Tinti, E., M. M. Scuderi, L. Scognamiglio, G. Di Stefano, C. Marone, and C. Collettini. 2016. "On the Evolution of Elastic Properties during Laboratory Stick-Slip Experiments Spanning the Transition from Slow Slip to Dynamic Rupture." *Journal of Geophysical Research: Solid Earth* 121(12):8569–94.
